# Mercury concentrations in biota in the Mediterranean Sea, a compilation of 40 years of surveys

**DOI:** 10.1038/s41597-019-0219-y

**Published:** 2019-10-16

**Authors:** S. Cinnirella, D. E. Bruno, N. Pirrone, M. Horvat, I. Živković, D. C. Evers, S. Johnson, E. M. Sunderland

**Affiliations:** 1 CNR-Institute of Atmospheric Pollution Research, Rende, Italy; 2 Jožef Stefan Institute, Ljubljana, Slovenia; 3 Biodiversity Research Institute, Portland, ME USA; 4 Harvard John A. Paulson School of Engineering and Applied Sciences, Harvard University, Cambridge, MA USA

**Keywords:** Environmental sciences, Ocean sciences

## Abstract

The Mediterranean Region has a long lasting legacy of mercury mining activities and a high density of sub-marine volcanoes that has strongly contributed to its mercury budget. In the last forty years, there have been recorded increases in mercury concentrations in biota that have spurred a growing number of research activities to assess the impact of mercury pollution on human health and environment. Field investigations that quantify mercury concentrations in marine biota have led to a large amount of experimental data scattered in many peer-reviewed publications making it difficult for modelling applications and regional environmental assessments. This paper reviews existing peer-reviewed literature and datasets on mercury concentration in marine flora and fauna (Animal, Plants and Chromista Kingdoms) in the Mediterranean basin. A total of 24,465 records have been retrieved from 539 sources and included in Mercury in Mediterranean Biota (M2B). Well-defined specimens account for 24,407 observations, while a few records include generic plankton and unidentified fish species. Among all considered species, we selected *Diplodus sargus*, *Sardina pilchardus*, *Thunnus thynnus* and *Xiphias gladius* to show trends of mercury concentration against WHO and EU limits. Few notes on how M2B is intended to support the implementation of the Minamata Convention on Mercury by a user-driven Knowledge Hub are finally reported.

## Abstract (summary)

**Table:** 

Measurement(s)	mercury
Technology Type(s)	digital curation
Factor Type(s)	geographic location • fishing area • species • sampling time • depth • weight • tissue
Sample Characteristic - Organism	Eukaryota
Sample Characteristic - Environment	ocean biome
Sample Characteristic - Location	Mediterranean Sea • Black Sea

Machine-accessible metadata file describing the reported data: 10.6084/m9.figshare.9886004

## Background & Summary

Harmful impacts of mercury on ecosystems and human health, including severe impacts on the central nervous system, were highlighted in Minamata Bay in 1953. By 1973, the 2nd Minamata Disease Research Group suggested that there might be chronic effects associated with mercury exposure through fish consumption^1^. This galvanized the scientific community to study diverse incidents of mercury pollution and led to the publication of numerous articles and reports in peer-reviewed literature. This has also been the topic of many conferences held over the last two decades^2^.

Emissions of anthropogenic mercury, transport through the atmosphere, deposition into ocean, subsequent transformation into methylmercury, and its incorporation into the marine food webs represent important components of the global mercury cycle. Over the past two decades, researchers have described many processes that affect methylmercury bioaccumulation and biomagnification^3^.

Mercury pollution is globally distributed but elevated biological concentrations of methylmercury are mainly found in seafood consumers and wildlife such as fish^4,5^, birds^6^, and marine mammals^7^. Methylmercury exposure is associated with neurotoxicity in humans and impacts the behaviour, physiology and reproductive success of wildlife^7^.

Field investigations assessing mercury concentrations in marine biota have been conducted in all oceans and seas^8^, including the Mediterranean since the 1970s. With the launch of the Mediterranean Action Plan (MAP) in 1975 as part of the UNEP’s Regional Seas Programme, the number of studies related to methylmercury exposure in marine biota and its impact on human health increased significantly^9^. The literature shows that mercury concentration in some fish species (with the same size range) observed in the Mediterranean Sea have concentrations that are several folds higher than those found in the Atlantic Ocean^10–12^. However, recent studies demonstrate that mercury concentrations in Mediterranean waters (about 1 pmol L^-1^) are similar to those measured in the adjacent North Atlantic Ocean^13–15^.

Over the past 40 years, this scientific investment has contributed to new analytical and modelling techniques for quantifying spatial and temporal patterns of mercury in different biotic and abiotic matrices. This, in turn, permits regional scale assessments of mercury contamination on human health and the environment. Although the Mediterranean Sea has been the focus of many studies, there are still gaps in our understanding of physical, chemical and climate processes that affect the dynamic of mercury compounds and changing direct and indirect releases of mercury from anthropogenic and natural sources^16,17^.

Within the GMOS project (http://www.gmos.eu/) and under the Global Observation System for Mercury (GOS^4^M) Flagship (http://www.gos4m.org/) of the Group on Earth Observations (GEO) (http://www.earthobservations.org), a wide literature survey was conducted to provide a comprehensive database for advanced assessments. The Mercury in Mediterranean Biota (M2B) database was compiled with publicly available records of mercury concentrations in marine biota reported in scientific papers, technical reports, national databases and meeting summaries, covering different spatial and temporal scales. This paper reports the methodology adopted to compile M2B and the advanced web services established to share information. It also addresses several key points for policy-makers to support their use of this resource.

## Methods

### Data sources and dataset compilation

The approach that was adopted to compile this database included the collection of mercury concentrations in biota, harmonization of information, verification and definition of a unique taxon, assignment of geographic location where not available and control of overlapping data. The specific methodology adopted for the compilation of M2B database has been structured through four different steps: document collection, duplication check, archiving and integration of additional information.

The information used to construct M2B has been retrieved from scientific papers in peer-reviewed journals, books, technical reports, Bachelor, Masters, and PhD theses, and project reports. The comprehensive commented list of references considered in this work is available at the same repository of the dataset^18^. Most documents are written in English but a few are in French, Spanish or Italian. In a few cases, data were downloaded from available datasets. A list of references was compiled and each document was coded with a unique number. Where necessary, notes were associated with the reference list. Published datasets were thoroughly checked with raw or unpublished data to avoid duplication and redundancy.

A number of parameters were selected (Table 1) from each document and manually archived into a spreadsheet. Dataset fields were compiled with existing information, whereas they were derived from figures when unavailable otherwise. Mercury concentrations were obtained from tables and granulated to the extent possible. In several cases, concentration data were reported as averages among individuals and, when available, minimum, maximum, standard deviation and sample size were included. Different mercury species, tissues (Table 2), sex, length, weigh, sampling depth, etc. were also collected.

**Table 1:** Description of collected parameters. List of collected parameters, definition and examples.

Name	Definition	Example
ID	Unique identifier	8132
Country	The jurisdictional water were sampling has been collected	GR
Loc	The specific location of sampling reported in the document	Crete
FAO_region	Code of FAO fishing area	37.3.1
Lat	Latitude (decimal degrees) of the site	35.006937
Lon	Longitude (decimal degrees) of the site	24.528896
Precision_code	The accuracy of geographical coordinates	2
Kingdom	Taxonomic group of highest hierarchical level	Animalia
Class	Taxonomic Class	Elasmobranchii
Order	Taxonomic Order	Carcharhiniformers
Family	Taxonomic Family	Triakidae
Specy_name	Scientific Gender and Species of the organism	*Mustelus mustelus*
Specy_com	Common name of the organism	Smooth-hound
Specy_code	Code of the specie: three first letters of the Genus and species	Musmus
Trophic level	Decimal number of position that an individual occupies compared to the basic trophic level represented by autotrophs	3.92
TL_ref	Trophic level obtained from different source	FB
Depth_m	Depth of sampling and unit of measure	-385
Lenght_cm	Length of sample and unit of measure	61.2
Weight_g	Weight of sample and unit of measure	518
Age_y	Age of fish and unit of measure	5
Sex	Sex of organism	F
Tissue_cod	Code to identify the studied tissue	WH
Hg_species	Specie of mercury analysed	HgT
DW_FW_WW	Water content of the sample as reference to mercury	FW
Mea_ug/kg	Mean mercury concentration and unit of measure	310
Min_ug/kg	Minimum mercury concentration and unit of measure	220
Max_ug/kg	Maximum mercury concentration and unit of measure	530
SD_ug/kg	Standard deviation of mercury concentration and unit of measure	100
SE_ug/kg	Standard error of mercury concentration and unit of measure	5
Sample_size	Number of samples	10
Reference	Reference to the original document reporting the data	Kousteni *et al*. 2006
Ref_n	Progressive number of the reference	212
Day	Day of sampling	25
Month	Month of sampling	06
Year	Year of sampling	1988
Remarks	Optional remarks on the record	Orig.:

**Table 2:** Tissue codes. Codes of tissues analysed to which mercury concentration data are referred to in M2B.

Code	Animal and Chromista tissue
AB – AR	Abdomen (Crustaceans) – Arms (Cephalopods)
BB – BL – BU – BM – BO –BR – BW	Backbone – Blood – Blubber – Bone – Bone –Brain – Body wall
CA – CH – CN	Carapace – Chelipeds – Central nervous system
DG – DT – DO – DW	Digestive gland – Digestive tract – Digestive organ – Digestive wall
EG – EM – EO – ES – EY	Egg – Embryo – Electric organ – Embryo sac – Eyes
FA – FI – FL – FO	Fat – Fillet (Fish) – Flesh – Foot (Gastropods)
GI – GO – GU	Gills – Gonads (sex indeterminate) – Guts
HA – HE – HP	Hatchling – Heart – Hepatopancreas
IN	Intestine
KD	Kidney
LI – LU	Liver – Lung
MA – ME – MI – MT – MU – MUD – MUL – MUO – MUT – MUV – MUW	Mantle – Melon – Milk – Mantle – Muscle (mixed, undetermined) – Muscles dark – Muscles of legs – Muscles dorsal – Muscles of tail – Muscles ventral – Muscles white
OV	Ovary
PA – PI – PL	Pancreas – Pincer (Crustaceans) – Placenta
RS	Residuals of tissues
SC – SH – SK – SO – SP – ST – STC	Scale(s) – Shell – Skin – Soft Part (Whole body without carapace or shell) – Spleen – Stomach (empty) – Stomach content
TE	Testicles
UT	Uterus
VI	Viscera
WH	Whole body
YA	Yolk and albumen
**Code**	**Plant tissue**
BAS – BLA	Basal Part – Blade of adult leaves
DLT	Distal Leaf Tip
EPI	Epiphytes
FRO	Frond
GRS	Green Segment above the Basal Part
LEA	Leaf green part
RHD – RHI – ROO	Rhizoid – Rhizome – Root
SCA – SHE – SHO – STO	Scales, remnant leaf sheaths – Sheath of adult leaves – Shoots – Stolon

A particularly unique case that was included is biomarker organisms. For example, transplantation techniques are frequently used to assess mercury contamination with mussels. This technique entails measuring mercury concentrations in individuals grown at an uncontaminated location (reference site), followed by transplantation to a contaminated site. In the case of transplantation, temporal trends, pollution gradients and coupled approaches (e.g. chemical and biological measurements) can provide integrated evaluation of the impact of pollution^19^. Information on the same individual before and after transplantation was included in the database. Data granularity (i.e., the lowest level of information) was the principle that has driven data archiving.

Geographic coordinates (latitude/longitude) for data collection are very important and are frequently not reported in older documents. In such cases, geolocations were assigned based on the description of locations or by means of a digitizing technique associated with the georeferencing process that was used to infer coordinates from geographical maps. The methodology adopted to assign geographical coordinates led to the definition of a precision code (Table 3).

**Table 3:** Geographical coordinates accuracy. Codes assigned to scale the accuracy of geo-referenced data provided.

Location Precision	Provided Location Info	Description
1	Specific latitude/longitude	Exact latitude/longitude provided in literature.
2	Specific description	Able to approximate latitude/longitude with specific site location information; match geographic features on provided map with Google Earth, or name of small area is given (e.g. city, small bay, cove) in literature.
3	Less Specific: <250 km	No latitude/longitude, or specific site location information as described above. Provided with a water body title (i.e. sound, bay, gulf, etc.) or description of region with a distance across of <250 km.
4A	Specific: <500 km	Latitude/longitude or specific site location information given for multiple sites, with single mean Hg value reported across all sites. Furthest sampling locations are <500 km apart.
4B	Vague: <500 km	No latitude/longitude, or specific site location information. Estimated sampling area based on literature description is <500 km across.
5A	Specific: <1,000 km	Latitude/longitude or specific site location information given for multiple sites, with single mean Hg value reported across all sites. Furthest sampling locations are 500–1,000 km apart.
5B	Vague: <1,000 km	No latitude/longitude, or specific site location information. Estimated sampling area based on literature description is 500–1,000 km across.
6A	Specific: >1,000 km	Latitude/longitude or specific site location information given for multiple sites, with single mean Hg value reported across all sites. Furthest sampling locations are >1,000 km apart.

To enrich the dataset, taxon were reported for all records and common names were checked against online resources. In a few cases, reported names were updated to the most recent nomenclature system. Primary online resources that were used to check common and the scientific names were:

- FishBase, a Global Information System on Fishes that includes 34,000 Species^20^;
- SealifeBase, a Global Information System on Marine Organisms that includes 76,300 Species^21^;
- World Register of Marine Species (WoRMS), a database on marine biota that includes 226,703 accepted species (http://www.marinespecies.org)^22^.

These sources were also used to derive additional information (e.g., the trophic level). Finally, several documents did not report details on the sampling date. In such cases, the data were time stamped as happening two years prior to the publication date, assuming that the two-year time interval is the average time necessary to sample, analyse, describe, and publish a dataset.

In order to gain insights into mercury bioaccumulation by species, the Mediterranean Basin was split in different FAO Divisions. In addition, a few North-South and East-West sections were established to analyse areal distributions of samples and the geographical context. We adopted such Divisions because they reflect EU rules on information that must accompany fishery and aquaculture products sold to consumers. These rules state that catch or production area is a mandatory information to be displayed for consumers. Therefore, an understanding of FAO Divisions associated with the collection of each sample can assist in the development of educational tools for consumers as a future application of this database.

### Database structure

M2B is structured in PostGIS [https://postgis.net/], an extender of PostgreSQL object-relational database and runs on a single host. It has a logical container for database objects and a logical container for Geographical Information System (GIS) objects. In the case of M2B spatial objects are represented by points, which include a spatial referencing system identifier (SRID). Selected SRID of information is 4326 or World Geodetic System 1984 (WGS84) coordinates (Latitude and Longitude in decimal degrees). To avoid excessive granularity of the database objects (tables) and considering available specimens, 118 objects were created (one for each specimen family).

The database also includes a description (metadata) of each object following the formal ISO 19115-1:2014 scheme. Each metadata point provides information about the identification of the object, the spatial extent, the data quality, the temporal consistency, the spatial reference, and other properties of digital geographic data and established services.

## Data Records

### Geographical and temporal overview

A total of 24,465 records retrieved from 539 sources were included in M2B. The full database is available at PANGAEA repository^18^. Well-defined specimens account for 24,407 observations, while few records include generic plankton or unidentified fish species. Mercury concentrations were mainly observed within territorial seas less than 12 nautical miles from the coast, as established by EU Directives.

**Fig. 1:**
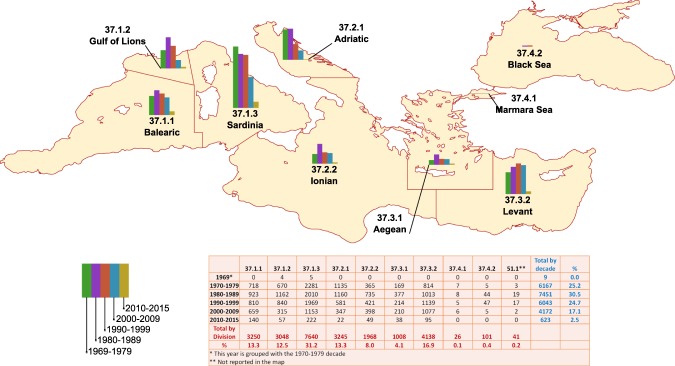
Effort of Countries over forty years of biota monitoring. Number of samples classified by decade. Distribution of samples (totals and percentages) in different FAO Divisions classified by decade.

Across FAO fishing division, the Sardinia Division (37.1.3) accounts for 31.2% of the total samples included in the database. The Marmara Sea (37.4.1) and Black Sea (37.4.2) account for less than 1% of the samples. The distribution of samples among other Divisions is detailed in Fig. 1. If we consider all the collected documents, countries that have made the most extensive field surveys include: Italy (39%), France (15%), Israel (13%), and Spain (11%). In 1969, Italy and Greece made their first survey. The fewest document were published between 1991–2000 compared to other periods. The year with the highest number of documents is 1985. For the Mediterranean waters of African Countries, few data were documented.

Most data records report total mercury (HgT) in biota. This reflects the sum of inorganic mercury and methylmercury (MeHg) in tissue. In a few cases a generic organic mercury was detected and reported in the dataset. For our collection of information, units of measure were harmonized by converting concentrations in mg kg^−1^, mg g^−1^, ng mg^−1^, ng g^−1^ and ppm to concentrations in μg kg^−1^. No other transformations were applied to the dataset. To harmonize measurements of mercury concentration in tissues with different water content (ww = wet weight; dw = dry weight), conversion factors obtained from literature were adopted. Details are reported in Supplementary Table 1.

### Monitoring programmes in the Mediterranean

Increasing scientific attention to environmental pollution and its threats to human and environmental health resulted in an increase in monitoring activities by national agencies.

The French national network for the observation of the marine environment (National Marine Environment Monitoring Network - Réseau National d’Observation, RNO) was established with the objective of assessing levels and temporal trends in contaminants as well as other seawater quality parameters^23^. All RNO activities started in 1974 were coordinated by the French Research Institute for the Exploitation of the Sea (IFREMER) and emphasized bivalves from the French shore.

The Italian database for the Sea Defense System (Sistema Difesa Mare, Si.Di.Mar.) was established in 1990 for the Adriatic Sea and was expanded in 1996 to the entire Italian shore. Mercury in water, sediment and biota is monitored by regional agencies.

The MED POL is the operational programme of the Mediterranean Action Plan (MAP). It was established in 1975 to assist Mediterranean Countries to implement three major protocols of the Barcelona Convention^24^. Monitoring activities are an essential component of the programme for tracking the efficiency of policy measures taken to reduce and control the level of pollution as well as to assess the status of the marine environment. MED POL Phase III published a database on the UNEP/MAP web site in 2010 that is no longer available. Observations related to mercury in biota from the MED POL database were integrated in M2B.

### Evolution of sampling methodologies and techniques of analysis

The example reported in Supplementary Table 2 clearly illustrates that methodological differences are primarily related to sample collection and analytical methods. For example, most studies reported limited information on the exact locations of sample collection and fish characteristics (e.g., specimen sex). Major differences in mercury concentrations can be related to differences in the sample preparation phase (drying and digestion) and dilution ratios for acid digestion. However, these details are often not provided in older studies. In addition, accuracy control and statistical methods used for data analysis differ across studies. In most recent papers, analytical accuracy was checked by regular analysis of certified reference materials and statistical analysis by means of ad-hoc software. Horvat *et al*.^25^ describes the benefits of the technological shift enabled by computers, software and new methods for statistical data analysis. While older papers are based on simple statistics, editorial policy today allows paper publication only if robust statistical analyses are conducted^26^.

Metadata information associated with M2B database shows that the most common methods used in the determination of mercury concentrations in different biota samples include: CVAAS (cold vapour atomic absorption spectroscopy), CVAFS (cold vapour atomic fluorescence spectroscopy), TD (direct analysis by thermal decomposition), ICP-AES (inductively coupled plasma-atomic emission spectroscopy), and ICP-MS (inductively coupled plasma-mass spectrometry).

Since its first development, CVAAS became the reference method for mercury determination and the US Environmental Protection Agency (EPA) adopted this technique and defined well established standards (e.g., EPA method 245.1). CVAFS has a better detection limit than CVAAS and a wider dynamic range. The US EPA has promulgated methods for mercury determination for CVAFS (e.g., EPA method 245.7). Over the last several decades, the quality of measurements has greatly improved due to the availability of suitable reference materials, calibration standards, and regular organization of inter-laboratory intercomparison exercises.

We generally do not observe differences in reported concentrations of mercury in fish samples obtained using different sampling methods, sample preparation procedures, and analytical methods/techniques. Differences in fish habitat, fish age/size, and the possibility of variable environmental mercury loadings make an exact assessment difficult without detailed modelling. A recent review of mercury speciation in the Adriatic biota showed no significant differences in fish mercury with time^27^. Possible sample contamination and the use of mercury-contaminated acids for sample digestion might have caused errors in older studies since results were often not corrected for blanks (this information is not commonly reported in older studies). Better sensitivity of some methods over the others (e.g. CVAFS over CVAAS) is not expected to result in large differences in reported mercury concentrations because they are generally high in fish samples.

Imprecision across measurements is a greater problem for comparing trends in biota with lower mercury concentrations (e.g. zooplankton). Mercury concentrations have been decreasing in the Adriatic zooplankton with time^27^. However, the use of different sampling methods, sample preparation procedures, and analytical methods does not allow for an assessment of contributing factors in this small dataset. In addition, the concomitant closure of chlor-alkali plants in the Adriatic and the Mediterranean Sea caused a corresponding decline in environmental mercury concentrations^28^. Assessing the factors contributing to the observed drop in zooplankton mercury concentrations is therefore difficult. Such problems motivated the development of this expanded dataset, which is much more detailed than previous work and will be used for future modelling.

### Insight Data

#### Trends by category

**Fig. 2:**
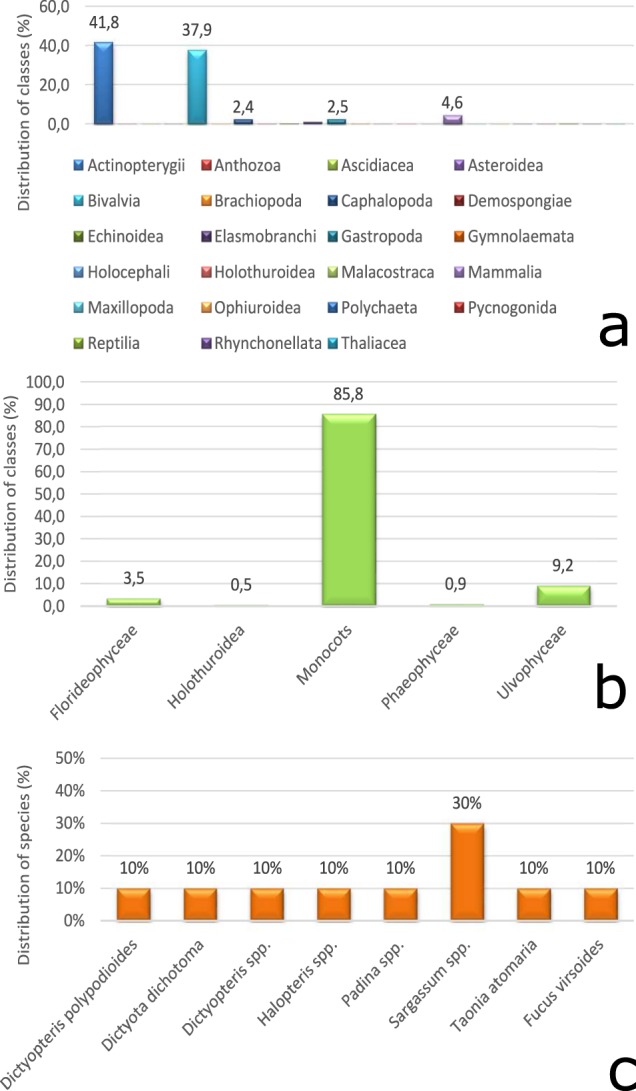
Representation of biota samples within the three Kingdoms in the Mediterranean. Distribution of classes within Animalia (**a**) and Plantae (**b**) Kingdoms. Phaeophyceae is the unique Class recorded for Chromista (**c**) Kingdom therefore only distribution of species is reported.

Animalia, Chromista and Plantae are the Kingdoms represented in M2B. In the case of Animalia, the largest number of observations pertain to Actinopterygii (41.8%) and Bivalvia (37.9%) (Fig. 2). Among Plantae, *Posidonia oceanica* (Monocots) are the most sampled specimens (87.1%) due to abundance over all seasons, capability to absorb pollutants, and ease of sampling^29–31^. Chromista is the least represented Kingdom, including the Class of Phaeophyceae. *Sargassum* spp. represents 30% of the total records.

The Mediterranean dataset is characterized by 22,749 records of total mercury (HgT) and 1,278 of methylmercury (MeHg) in 394 species. In a few cases, records report Hg(II) (4) and organic mercury HgO (434), which probably refers to MeHg even if the analytical procedure is not available to see exactly what was determined. Data are limited to only a few marine eco-regions and are mostly concentrated in the northern part of the Mediterranean, where polluted areas prevail^27,32^. Data are limited in the southeastern part of the Mediterranean Sea for two reasons: (1) fewer research/monitoring efforts in Countries facing the Sea, and (2) possible publication of datasets in national languages that are not known by the Authors.

The distribution of sampled organisms living in specific marine habitats is not uniform in the dataset. Benthic organisms from neritic habitats are the most highly represented group. As expected, there are a large number of Mediterranean mussel (*Mytilus galloprovincialis*) samples, which are frequently used as a favoured bioindicator^27,33,34^.

**Fig. 3:**
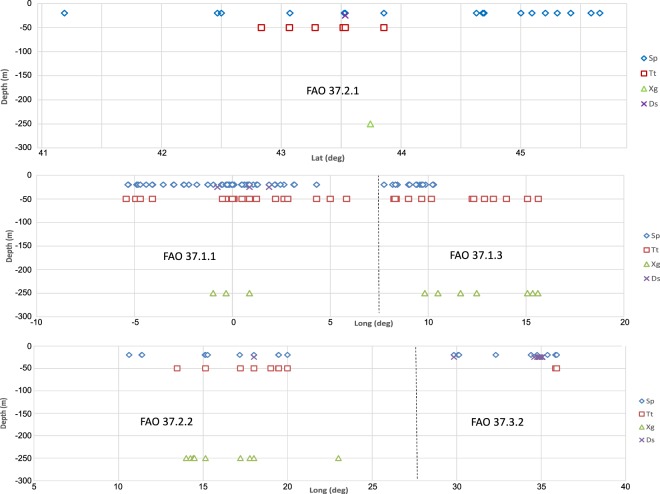
Distribution of selected species in the Mediterranean. Distribution of *Sardina pilchardus* (Sp), *Thunnus thunnus* (Tt), *Xiphias gladius* (Xg) and *Diplodus sargus* (Ds) along sections of Adriatic Sea (A), Western Mediterranean Sea (B) and Central-Eastern Mediterranean Sea (C). See Fig. 5 for details on sections.

The distribution of *Sardina pilchardus* (Sp), *Thunnus thynnus* (Tt), *Xiphias gladius* (Xg) and *Diplodus sargus* (Ds) has been considered in relation with depth of sampling and in different marine regions (Fig. 3). As expected, small organisms were sampled near the sea surface (i.e., Sp and Ds), while the largest ones were sampled at depth of common occurrence (i.e., Tt and Xg). Sp was regularly sampled in different marine regions, followed by Tt, which is under-represented in FAO Division 37.3.2. In addition, the ecological behaviour of species affects sampling in different marine regions. For example, swordfish were not often sampled because they are primarily found in deeper oceanic waters and colder marine environments^20^, which limits sample collection during most research oceanographic cruises.

**Fig. 4:**
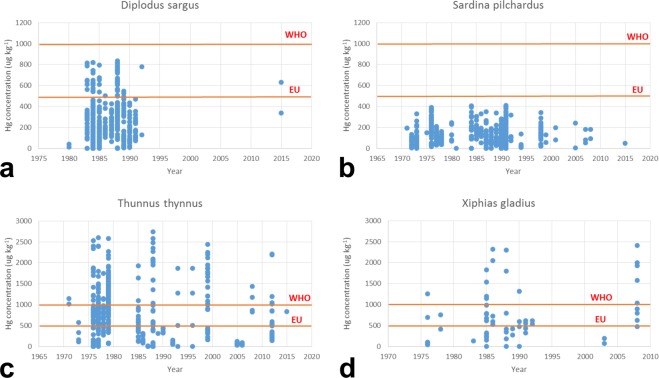
Trends in mercury concentrations and comparison to EU and WHO limits. Examples of concentration trends for White seabream (**a**), European pilchard (**b**), Atlantic bluefin tuna (**c**) and Swordfish (**d**). EC and WHO established mercury consumption general guideline level of 0.5 mg kg^−1^ and 1.0 mg kg^−1^, respectively. Concentrations include only HgT in tissues (see Table 1 for specification).

Trends reported in Fig. 4 show that organisms with the highest mercury concentration are Cetartiodactyla (arithmetic average of 117.2 ug g^−1^ ww), followed by Lophiiformes (9.1 ug g^−1^ ww) and Squaliformes (4.3 ug g^−1^). Comparing these trends with European Commission (EC) and World Health Organization (WHO) limits, for White seabream (*Diplodus sargus*) (Fig. 4a), European pilchard (*Sardina pilchardus*) (Fig. 4b), Atlantic bluefin tuna (*Thunnus thynnus*) (Fig. 4c) and Swordfish (*Xiphias gladius*) (Fig. 4d) it was found that HgT are lowest in smaller, short-lived fish and always below EC (2002) and WHO (2010) general guideline level of 0.5 mg kg^−1^ ww and 1.0 mg kg^−1^ ww, respectively^35,36^. Conversely, highest HgT concentrations were found in large, long-lived species such as pelagic marine species like tuna and swordfish.

The most abundantly analysed tissues in biological sample were the soft tissue for Bivalvia (34%) and muscle for Animalia (15%). Tissues from fish and mammals as fillets and the abdomen and whole body samples, account for 22%, 4.4%, 11.3% of all samples, respectively. Mercury concentrations in small species are usually measured after homogenization of the whole fish, which includes some tissues that might be enriched in Hg compared to muscle^37^. Whole tissues are frequently sampled in algae and molluscs.

**Fig. 5:**
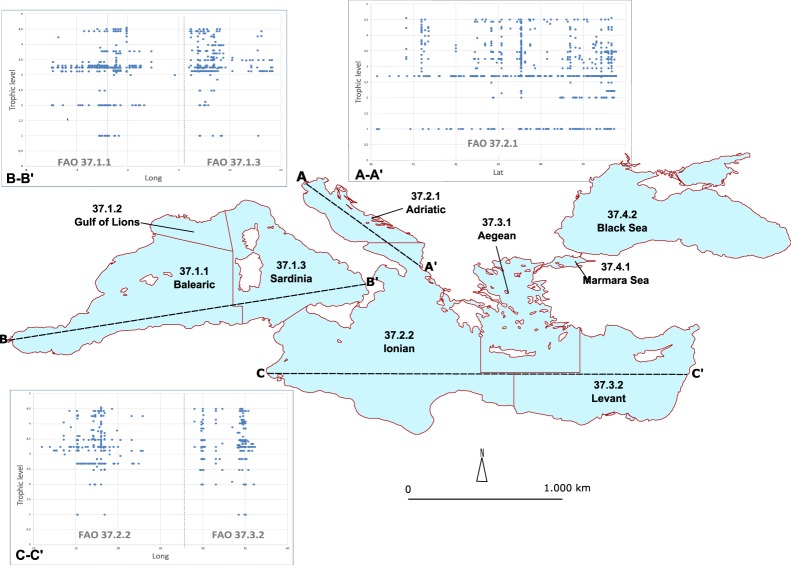
Distribution of records by trophic levels within different sectors. Trophic levels profiles along the North to South in the Adriatic Sea (A-A’), West to East in the Western Mediterranean Basin (B-B’) and the Central-Eastern Mediterranean Sea (C-C’).

To analyse the spatial distribution of Hg bioaccumulation by Trophic Levels (TLs), the Mediterranean region was split in different sections as follow: Section A spans from NW to SE of the Adriatic Sea (38°55′N to 45°46′N); Section B from the Western Balearic Sea to the Southern Tyrrhenian Sea (5°28′W to 16°13′E); and Section C from Tunisian to the Israeli coast (9°52′E to 36°08′E) (Fig. 5).

Samples of organisms at TL 1 (algae) and TL 2.69 (mussel) were uniformly distributed along section A whereas they were scattered along sections B and C. Considering the available records for recognized trophic nets due to the limited number of represented TLs and organisms, only few locations in each FAO Division were potentially suitable for performing a biomagnification analysis.

Recognized trophic nets by considering records availability are those reported in Supplementary Table 3. A very preliminary analysis of M2B data shows that not all FAO Divisions and all years provide data that can be used to perform a detailed analysis aiming to assess the degree of mercury bioaccumulation by species and by eco-region.

### M2B in support of policy makers

The M2B database will make available key field data that may be used by the modelling community to better characterize the relationships between atmospheric deposition to surface marine waters and mercury bioaccumulation in biota. The ongoing debate among experts and policy makers regarding the implementation of a Global Monitoring Plan for assessing mercury in environmental matrixes (i.e., primarily air and biota) and risk associated to human exposure, requires evaluated models that can be used by policy makers to perform a preliminary assessment of the effectiveness of measures that nations can undertake to achieve the target of the Minamata Convention on Mercury (MCM)^38,39^. Therefore the M2B is intended to support the development of evaluated marine models as part of a user-driven Knowledge Hub that can be used to assess the relative importance of different mercury sources (anthropogenic or natural-driven sources) on human health at regional (e.g., reference to FAO eco-regions) and global scale.

Additional tools that can be developed that support decisions of seafood consumers. Understanding average concentrations of mercury in seafood from a specific FAO Division can help estimate of mercury intake by consumers, including vulnerable populations like pregnant and breastfeeding mothers, as well as infants and young children. Such tools can help individuals make informed choices when choosing seafood that is nutritious and safe to eat.

Additionally, the following key-points can be addressed with the support of M2B:

- Understanding the relationship between trends in atmospheric deposition to surface marine waters and mercury bioaccumulated in marine biota;
- Evaluating mercury concentration trends in different fish catch/production FAO Divisions, allowing decision makers on limitation of fish catch in more polluted areas;
- Assessing the biomagnification process to help consumer decisions on seafood with low level of mercury intake.

## Technical Validation

Technical validation of this database was performed at two levels related to geographical information control and the quality of measurements.

Geographical information defines the geographical location of the sampling. Unfortunately, many peer-reviewed studies and technical reports published in the 1980s and early 1990s provide only generic information on the study areas and sometimes a low-resolution geographic map. Therefore, in those cases, the geographical information was inaccurate and incomplete and LAT-LON coordinates were determined though a best technical estimate. However, recent studies provide very accurate geographic information thanks to use of GPS sensors integrated in tablets and smartphones, that allowed research groups to link their sampling locations to geo-referenced maps. In order to scale up the accuracy of geographic information reported in M2B a precision code was assigned to each site/location for which data were reported.

Table 3 describes all the codes from 1 to 6 assigned starting from the simplest case where exact latitude/longitude was provided, to those case that only generic information or low resolution maps were provided.

## Usage Notes

M2B is also archived in the Global Observing System for Mercury (GOS^4^M) Spatial Data Infrastructure (SDI). GOS^4^M is a Flagship of the Group on Earth Observations (GEO) (https://www.earthobservations.org), which is aimed to:

- increase the availability and quality of Earth Observation (EO) data and information to contribute to the tracking of mercury released to the global environment;
- harmonize metadata production, archiving and sharing data on mercury in the environment;
- develop advanced web services in support of policy mandate through the MCM.

The infrastructure was designed to bridge the gap between EO data and users through a Community Portal based on the Lave and Wenger (1991). The M2B SDI has been structured in three different logic levels: the Data Storage Layer (DSL), the Business Logic Layer (BLL), and the Application Layer (AL)^40^. A Database Management System (DBMS), which stores metadata, observations and functional data (e.g. users’ roles, credentials on datasets) represents the DSL and is accessed by BLL components that perform metadata editing, data management, map creation, and data dissemination. Among such components, a map server is used to export data through Open Geospatial Consortium (OGC) compliant services while the metadata server is used to manage metadata and its related catalog. Server components export Web Services such as Web Feature (WFS), Web Map (WMS), Web Coverage (WCS) and Catalogue Services for the Web (CS-W), through the Hypertext Transfer Protocol (HTTP).

In the case of M2B, it can be discovered through the GOS^4^M Geoportal (http://www.geoportal.org/community/gos4m) (keyword: biota). The discovered resource provides links both to data and metadata.

Comparison among observations must be carefully investigated considering that methodologies of analysis and instruments detection have changed over the years.

## Supplementary Information

Supplementary information

## Data Availability

The dataset was compiled within a spreadsheet and exported in the Comma Separated Value (CSV) format. No additional codes were used.
