# Supplementary material for: Mercury concentrations in biota in the Mediterranean Sea, a compilation of 40 years of surveys
Source: Sci Data. 2019 Oct 16;6:205. doi: 10.1038/s41597-019-0219-y (PMC6795892; doi:10.1038/s41597-019-0219-y)
Supplement: Supplementary file 1 — Supplementary information [file 41597_2019_219_MOESM1_ESM.docx]

**Supplementary Table 1. Conversion factors from Dry Weight (DW) to Fresh Weight (FW).** Conversion factors adopted to convert DW to FW for each Taxon and related reference.

| **Taxon** | **DW-to-FW conversion factors** |
| --- | --- |
| Actinopterygii | 5 |
| Ascidiacea | 6.3 |
| Asteroidea | 32.9 |
| Bivalvia | 6 |
| Branchiopoda | 20 |
| Cephalopoda | 20 |
| Demospongiae | 16.8 |
| Echinoidea | 3.5 |
| Elasmobranchii | 5 |
| Florideophyceae | 6 |
| Gastropoda | 7.5 |
| Holothuroidea | 19.3 |
| Malacostraca | 17 |
| Mammalia | 3 |
| Maxillopoda | 16.5 |
| Monocots | 6 |
| Ophiuroidea | 47.1 |
| Phaeophyceae | 6 |
| Polychaeta | 19.8 |
| Reptilia | 5 |
| Thaliacea | 10 |
| Ulvophyceae | 6 |
| Phytoplankton | 20 |
| Zooplankton | 10 |
| *DW to FW conversion factors were obtained from Cossa et al. 2012, Cresson et al. 2014, Dietz et al. 1996, Knauer and Martin 1972, McMahon et al. 2005, Nasci et al. 1998, Ricciardi and Bourget 1998, or were estimated based on the biological similarities with organism(s) for which conversion factors are known.* | |

**Supplementary Table 2. Comparison between 1978 and 2014 papers on mercury in biota.** For 2014 the methodology refers to total mercury detection.

|  | **Yannai and Sachs, 1978** | **Horvat et al. 2014** |
| --- | --- | --- |
| Collection | Along the entire Mediterranean coast of Israel between May 1974 and Sep 1975. Fishing depth, specimen, weight and size reported in tables. | From August to October 2005 in the central part of the Gulf of Trieste. A total of 5 specimens of eagle ray, 17 specimens of bull ray, 8 specimens of the pelagic stingray and a single specimen of the common stingray The collected ray species were identified at the species level and their size measured to the nearest millimetre. Gender was determined according to the presence of claspers. |
| Drying |  | Freeze-dried (Christ Alpha 1-4) over a period of 4 days at −40 °C and a pressure of 0.02 mbar |
| Homogenization | In a food blender | With a planetary micro-mill (Fritsch pulverisette 7) |
| Digestion | Aliquots of 0.5 to 1.0 g (wet weight) in 4 ml of nitric acid in a Teflon decomposition cell at a temperature of 130°C for 1 hr | 500 mg of sample was weighed directly in a 100-mL volumetric flask followed by the addition of 3 mL of HNO3 (65 %), 1 mL HClO4 (70 %) and, finally, 5 mL of H2SO4 (96 %). The vessels were closed and the mixture was left to react at room temperature for an hour. The vessels were then placed for 20 min on a hot plate at 230 °C. |
| Reduction to elemental form | Sample transferred to a 100 ml Erlenmeyer flask with the aid of 45 ml of water and stannous chloride | The digest was diluted with Milli-Q water. An aliquot of the digest was added to the reduction cell; after reduction with SnCl2, |
| Determination | Flameless atomic absorption spectrophotometry using a Coleman Mercury Analyser System | Mercury was swept from the solution by aeration and concentrated on a gold trap.  Mercury was then released from the gold trap by heating and measured on an LDC Milton Roy instrument.  Cold vapour atomic absorption spectrophotometry |
| Accuracy control | No control | Checked by regular analysis of certified reference materials (CRMs) certified for total Hg. The CRMs DORM-2 (dogfish muscle), DOLT-3 (dogfish liver) and TORT-2 (lobster hepatopancreas), obtained from NRCC and IAEA-350. |
| Limit of detection | LOD not reported. | The limit of detection (LOD), expressed as the SD of the blank, was 0.2 ng/g; the limit of quantification (LOQ) was 1 ng/g. |
| Statistical analysis | All the assays-for animals, [...] were performed in triplicate.  As not specified, it was assumed that simple averages were used to obtain final values. | Pearson’s correlation coefficients were used to calculate correlations among variables, and t test was used for the statistical significance of differences for THg [...] in male  and female tissues of the bull ray species using SAS/STAT software. |
| Citations | 10 | 67 |

**Supplementary Table 3. Main trophic nets in the Mediterranean.** Selected species are those analysed for mercury in tissues (Stergiou and Karpouzi, 2002).

| **Species** | **Main prey** | **Trophic Level** |
| --- | --- | --- |
| **a)** |  |  |
| *X. gladius* | Cephalopods – T. sagittatus | 4.46 |
| *T. sagittatus* | Mainly animals (TL 2,8 and up) - C. harengus | 4.40 |
| *C. harengus* |  | 3.23 |
| Copepoda |  | 2.00 |
| Benthic algae |  | 1 |
| **b)** |  |  |
| *S. dumerili* | Mainly animals (TL 2,8 and up) - B. boops - Sardina pilchardus | 4.06 |
| *B. boops* | Algae- Copepods | 3.11 |
| Benthic algae |  | 1 |
| **c)** |  |  |
| *Z. faber* | Mainly animals (troph. 2.8 and up) Zoobenthos- Nekton- S. porcus | 4.24 |
| *S. porcus* | Mainly animals (troph. 2.8 and up)- G. niger | 3.78 |
| *G. niger* | Mainly animals (troph. 2.8 and up) | 3.36 |
| Copepoda |  | 2.00 |
| Benthic algae |  | 1 |
| **d)** |  |  |
| *D. sargus* | Mainly animals (troph. 2.8 and up) – P. lividus | 3.24 |
| *P. lividus* | Algae | 2.41 |
| Benthic algae |  | 1 |
| **e)** |  |  |
| *E. marginatus* | Mainly animals (troph. 2.8 and up) – S. salpa | 4.08 |
| *S. salpa* | Plants- Benthic algae and Posidonia oceanica | 2.14 |
| *Posidonia oceanica* |  | 1 |

**Supplementary References**

1. Cossa D., Harmelin-Vivien M., Mellon-Duval C., Loizeau V., Averty B., Crochet S., Chou L. & Cadiou J.-F. Influences of Bioavailability, Trophic Position, and Growth on Methylmercury in Hakes (Merluccius merluccius) from North-western Mediterranean and North-eastern Atlantic. *Environ. Sci. Technol.* **46,** 4885–4893 (2012).
2. Cresson P., Fabri M.C., Bouchoucha M., Brach Papa C., Chavanon F., Jadaud A., Knoery J., Miralles F. & Cossa D. Mercury in organisms from the North-western Mediterranean slope: Importance of food sources. *Sci. Total Environ*. **497–498,** 229–238 (2014).
3. Dietz R., Riget F. & Johansen P. Lead, cadmium, mercury and selenium in Greenland marine animals. *Sci. Total Environ*. **186,** 67–93 (1996).
4. Knauer G.A. & Martin J.H. Mercury in a marine pelagic food chain. *Limnol. Oceanogr.* **17,** 868–876 (1972).
5. McMahon C.A., Long S., Ryan T.P., Fegan M., Sequeira S., Dowdall A., McKittrick L., Wong J., Hayden E., Murray M., Colgan P.A. & Pollard D. *Radioactivity Monitoring of the Irish Marine Environment 2002*. Radiological Protection Institute of Ireland, pp 44 (2005).
6. Nasci C., Da Ros L., Campesan G. & Fossato V.U. Assessment of the impact of chemical pollutants on mussel, Mytilus galloprovincialis, from the Venice Lagoon, Italy. *Mar. Environ. Res.* **46,** 279–282 (1998).
7. Ricciardi A. & Bourget E. Weight-to-weight conversion factors for marine benthic macroinvertebrates. *Mar. Ecol. Prog. Ser*. **163,** 245–251 (1998).
8. Yannai S. & Sachs K. Mercury compounds in some Eastern Mediterranean fishes, invertebrates, and their habitats. *Environ. Res.* **16,** 408–418 (1978).
9. Horvat M., Degenek N., Lipej L., Tratnik J.S. & Faganeli J. Trophic transfer and accumulation of mercury in ray species in coastal waters affected by historic mercury mining (Gulf of Trieste, northern Adriatic Sea). *Environ. Sci. Pollut. R.* **21,** 4163–4176 (2014).
10. Stergiou K. I. & Karpouzi V. S. Feeding habits and trophic levels of Mediterranean ﬁsh. *Rev. Fish Biol. Fisher.* **11,** 217–254 (2001)
